# Chemical Composition and Potential Biological Activity of Melanoidins From Instant Soluble Coffee and Instant Soluble Barley: A Comparative Study

**DOI:** 10.3389/fnut.2022.825584

**Published:** 2022-02-10

**Authors:** Sofia Antonietti, Amélia M. Silva, Cristiana Simões, Diana Almeida, Luis M. Félix, Adele Papetti, Fernando M. Nunes

**Affiliations:** ^1^Food and Wine Chemistry Lab, Chemistry Department, CQ-VR, Chemistry Research Centre – Vila Real, School of Life Sciences and Environment, University of Trás-os-Montes e Alto Douro, Vila Real, Portugal; ^2^Department of Drug Sciences, University of Pavia, Pavia, Italy; ^3^Department of Biology and Environment (DeBA-ECVA), University of Trás-os-Montes e Alto Douro, School of Life Sciences and Environment (UTAD-ECVA), Vila Real, Portugal; ^4^Centre for Research and Technology of Agro-Environmental and Biological Sciences (CITAB-UTAD), Vila Real, Portugal

**Keywords:** melanoidins, instant soluble coffee, instant soluble barley, phenolics, anti-inflammatory, antioxidant, anti-proliferative, fibre

## Abstract

In this work a comparative study of the chemical composition and potential biological activity of high molecular weight (HMW) melanoidins isolated from instant soluble coffee (ISC) and instant soluble barley (ISB) was performed. ISB HMW melanoidins were almost exclusively composed by an ethanol soluble (EtSn) melanoidin fraction composed by glucose (76% w/w) partially susceptible to *in vitro* digestion, whereas ISC was composed mainly by arabinogalactans (~41% w/w) and lower amounts of galactomannans (~14% w/w) presenting a range of ethanol solubilities and resistant to *in vitro* digestion. Melanoidins from ISC presented a significantly higher content of condensed phenolic compounds (17/100 g) when compared to ISB (8/100 g) showing also a higher *in vitro* scavenging of ABTS^•+^ (329 mmol Trolox/100 g vs. 124 mmol Trolox/100 g) and NO radicals (inhibition percentage of 57 and 26%, respectively). Nevertheless, ISB EtSn melanoidins presented, on average a higher inhibitory effect on NO production from LPS-stimulated macrophages. ISB melanoidins, up to 1 mg/mL, did not induce toxicity in Caco-2, HepG2 and RAW 264.7 cell lines while at the highest concentration ISC slightly reduced cell viability. Thus, consumption of a diet rich in ISC and ISB melanoidins may reduce the oxidative stress, the inflammatory levels and increase the protective effects against chronic inflammatory diseases.

## Introduction

Coffee is one of the most popular non-alcoholic beverages around the world with an estimated world annual consumption of 500 billion cups (1). Certainly, one of the factors for this popularity is its caffeine content, the best-known psychoactive stimulant (2), that improves cognitive performance (3). Coffee drinks contain, besides caffeine, thousands of other constituents, including carbohydrates, lipids, nitrogenous compounds, vitamins, minerals, alkaloids, and phenolic compounds (4, 5), some of them with potential health-beneficial properties. Chlorogenic acids, for example, are naturally antioxidants and their presence slow down the inflammatory process, thereby providing protection from the hazardous effect of free radicals and against endothelial damage (6). Debate still persists whether coffee is beneficial or troublesome for human health. Its consumption has been associated with a momentous decrease in chronic diseases such as Parkinsonism, diabetes mellitus, and several cancers (7–9). Consumption of coffee may inhibit inflammation and thus reduce the risk of cardiovascular and other inflammatory diseases in postmenopausal women (10).

On the other hand, people enjoying coffee flavour but being sensitive to caffeine have a range of coffee substitutes to drink, normally produced by roasting cereal grains like wheat, barley, malted barley, acorns or containing chicory and dandelion roots and figs (11). Among these coffee substitutes, roasted barley is one of the most consumed and also studied coffee substitute (12), although much less studied than coffee.

It has been shown that consumption of whole grain barley reduces the risk of developing chronic diseases (13, 14). These health benefits of whole barley consumption have been attributed to barley fibre, especially β-glucan (15). Nevertheless, whole grain barley also contains phytochemicals including phenolic acids, flavonoids, lignans, tocols, phytosterols, and folate (13, 14) that exhibit antioxidant, anti-proliferative, and cholesterol lowering abilities (16–19). Therefore, the presence of high concentration of these phytochemicals in barley may also contribute to its health benefits.

Due to the roasting process applied either to coffee or barley, melanoidins are formed and contribute for the brown colour of the roasted products. Melanoidins are the final products of the Maillard reaction and are defined as brown coloured, nitrogen containing, high molecular weight compounds (20). Coffee is the second most abundant source of melanoidin intake after bread (21). Considering the high intake of melanoidins as part of our daily diet, their biological activity and potential impact on human health are topics of great interest. Previous studies have looked into the biological activities that have been attributed to melanoidins, namely their antioxidant, antimicrobial, prebiotic, anti-cancer, and antihypertensive activities (22). Melanoidins, due to their biological activities, are thought as functional ingredients modulating multiple biological processes (23). Due to the presence of phenolic compounds in vegetable products subjected to roasting it has been shown that phenolic compounds can be present in melanoidins from different sources, adsorbed, ester linked, glycosidically linked or present in more complex structures, generally described as condensed structures (24–35). It has been shown that some of melanoidin's biological activity, as for example its antioxidant activity, may be linked to the presence of phenolic compounds in melanoidins structures, therefore, phenolic compounds linked to melanoidins structure can be a key structural feature of food melanoidins (23, 29, 30, 32–34). It is known that there is reduced intestinal absorption of indigestible melanoidin (36, 37). However, a few number of *in vivo* studies reporting the fate of ingested melanoidins refer that a small portion of ingested melanoidins are excreted in urine yet at very low amounts; as example, Homma et al., showed that rats fed with a diet supplemented with 2% (w/w) of non-dialysable, high-molecular-weight (HMW) melanoidin fraction, excreted ~26% in the faeces and about 1.8% in the urine (38). On the other hand, rats fed with a diet supplemented with low molecular weight (LMW) melanoidins (<10 kDa) showed higher percentage of excretion in urine (~27%) (39); showing that melanoidins are absorbed at intestinal tract, however absorption depends on molecular weight and solubility (40, 41). Apart from these studies, melanoidins are reported to exert most of its biological effects by modulating gut immunity (42–44).

Although in Western countries, with the exception of UK, homemade coffee drinks are mainly prepared with ground roasted coffee beans, in the emerging coffee markets and UK instant soluble coffee (ISC) is the preferred form of home consumption (45). Although fresh coffee is still dominant in the coffee industry, the ISC consumption is increasing worldwide driven by the convenience, versatility and a range of derived products, like those containing added sugar and cream (46). Taking into consideration the growing importance of ISC consumption and the fewer number of ISC melanoidin studies as well as the presence in the market of instant soluble coffee barley (ISB), whose chemical and biological properties are even less addressed (32, 47), the aim of the present work was to have a deeper understanding of the chemical structure of ISC and ISB melanoidins. For these purposes, we determined their chemical structure and studied their potential biological activities such as antioxidant, anti-inflammatory, neuroprotective, and anti-diabetic effects. The bioactivity of these melanoidins was evaluated by *in vitro* antioxidant methods and for the first time their cytotoxic effect was tested in two human cancer cell lines, namely Caco-2 and HepG2; in addition, the antidiabetic, antiaging, and neurobiological activities were evaluated *in vitro* by assessing their inhibitory activity against α-amylase, α-glucosidase, elastase, tyrosinase, and acetylcholinesterase (AChE).

## Materials and Methods

### Materials

Instant soluble coffee (Delta Company, Rio Maior, Portugal) and instant soluble barley (Crastan S.p.a., Pontedera, Italy) were purchased on the market.

### Chemicals

Ethanol, methanol, formic acid, acetic acid, sulphuric acid, hydrocloric acid, ethyl ether, urea, Folin-Ciocalteu's reagent (±)-6-hydroxy-2,5,7,8-tetramethylchromane-2-carboxylic acid (Trolox), potassium persulfate, 2,2′-azino-bis(3-ethylbenzothiazoline-6-sulfonic acid) diammonium salt (ABTS), sodium nitrite, sodium hydroxide, sodium carbonate, sodium molybdate, phosphoric acid, 2-deoxy-D-ribose, hydrogen peroxide solution, 2-thiobarbituric acid (TBA), 5,5′-dithiobis(2-nitrobenzoic)acid (DTNB), enzymes and reagents for all enzymatic activities were purchased from SIGMA Aldrich^®^ (Spain). Sodium nitroprusside, sulfanilamide, *N*-naphthylethylamide, trichloroacetic acid (TCA), arabinose, xylose, rhamnose, fucose, glucose, galactose, mannose, 2-deoxyglucose, veratric acid, catechin, gallic acid, 3,4-dihydroxybenzoic acid, 4-hydroxybenzoic acid, catechol, hydroquinone, zinc powder, caffeic acid, ferulic acid, 4-hydroxybenzoic, and ascorbic acid were purchased from Merck^®^ (Germany). Ethylenediamine tetraacetic acid (EDTA), sodium hydroxide, and aluminium chloride (III) were purchased from Panreac^®^ (Spain). Chlorogenic acid, was obtained from Extrasynthèse^®^ (France). Total Dietary Fibre kit (K-TDR-100A) was purchase from Megazyme (Ireland). Foetal bovine serum (FBS), Dulbecco's Modified Eagle's Medium (DMEM) (Gibco), L-glutamine, penicillin, streptomycin, and Alamar Blue were purchased from Invitrogen, Alfagene^®^ (Portugal).

### Isolation and Purification of Instant Soluble Coffee (ISC) and Instant Soluble Barley (ISB) Melanoidins

Melanoidins from ISC and ISB were isolated after dissolving 10 g ISC or ISB in 200 mL of water at 95°C. After stirring for 10 min, infusions were filtered through a size 2 sintered glass philtre and the remaining residue was washed with further 20 mL water. The filtrate was concentrated to approximately 50 mL by vacuum evaporation at 35°C and the solution was dialyzed at 4°C using a 12–14 kDa cut-off dialysis membrane (Medicell Membranes Ltd.; London, UK) with 10 water renewals. The retentate obtained was freeze-dried to obtained ISC and ISB high molecular weight material (HMWM).

### Fractionation of the HMWM by Graded Ethanol Precipitation

For ISC- and ISB-HMWM fractionation by graded ethanol precipitation, the method described by Nunes et al. (48) was used with some slight modifications. One *gramme* of HMWM was dissolved in 100 mL of a 6 M urea aqueous solution and stirred for 1 h at room temperature to totally dissolve the HMWM. Afterwards, 105 mL of 96 % ethanol was added and the mixture was cooled to 4°C and stirred for 1 h. The formed precipitated (Et50) was removed by centrifugation (24,400 × *g*, 20 min, 4°C). Another aliquot (210 mL) of ethanol 96 % was added and the mixture was stirred for 1 h at 4°C. The formed precipitate (Et75) was separated from the supernatant (EtSn) by centrifugation at 24,400 × *g* for 20 min at 4°C. The precipitates (Et50 and Et75) were suspended in 50 mL of water and dialyzed to remove the urea. EtSn fraction was concentrated by vacuum evaporation (Buchi, Germany) and then dialyzed as described above. After dialysis water was removed by freeze-drying.

### Carbohydrate Composition

The carbohydrate composition of ISC- and ISB-HMWM and of the fractions obtained by ethanol graded addition was determined after acid hydrolysis with 1 M sulphuric acid and analysed by GC with flame-ionisation detection after borohydride reduction and acetylation as described by Coimbra et al. (49) and quantified using the internal standard method (internal standard 2-deoxyglucose).

### Determination of Adsorbed, Ester-Linked and Condensed Phenolic Structures in ISC and IBC High Molecular Weight Material

For profiling the different phenolic compounds that can be associated with ISC and ISB melanoidins, namely, adsorbed, ester-linked or in condensed structures, the protocol described by Coelho et al. (28) was used. Briefly, for the determination of adsorbed phenolics, each HMWM containing melanoidins (5 mg/mL in 1 M NaCl) was analysed by reversed-phase HPLC (100 μL injection volume). For the determination of ester-linked phenolics, each HMWM was subjected to alkaline saponification (28) consisting in the addition of 750 μL 2 M NaOH solution containing 2% ascorbic acid (w/w) and 20 mM ethylenediaminetetraacetic acid (EDTA) to 750 μL of each HMWM solution (12 mg/mL in water). After incubation for 1 h at 30°C, the mixture was quenched to pH 1 with 330 μL of 5 M HCl and then stored for 2 h at 4°C. The precipitate was removed by centrifugation and the supernatant was analysed by reversed-phase HPLC. For the determination of condensed phenolics, 1 g of solid NaOH and 100 mg of zinc dust were weighed, and, after melting the mixture at 350°C in a nickel crucible, 5 mg HMWM were added. After 10 s, the nickel crucible was rapidly cooled in an ice bath. The fusion cake was solubilized by adding 6 M HCl, and then 200 μL of internal standard solution (1 mg/mL of veratric acid in ethanol) were added and acidified with 6 M HCl to achieve pH 1–2. The acidic mixture was extracted three times with 50 mL of diethyl ether. After evaporation of the organic solvent under vacuum, the residue was analysed by reversed-phase HPLC (29).

### Quantification of Total Phenols and *Ortho*-Diphenols

The total phenolic content of extracts was determined using the Folin-Ciocalteu method according to Silva, Ferreira and Nunes (50). A calibration curve was constructed using gallic acid and the results were expressed as mg gallic acid equivalents (GAE) per 100 g of fresh weight. The *ortho*-diphenol content was determined according to Soufi, Romero, and Louaileche (51) and the results were expressed as mg GAE per 100 g of dry weight, after the construction of a calibration curve using gallic acid.

### *In vitro* Antioxidant Activity Assessment

#### ABTS Radical Cation Decolorization Assay

For determining the ABTS [2,2′-azino-bis(3-ethylbenzothiazoline-6-sulphonic acid)] radical cation scavenging activity, the protocol of Silva, Ferreira and Nunes (46) was used with slight modifications. ABTS^•+^ radicals were produced by mixing ABTS with potassium persulfate (K_2_S_2_O_8_). The mixture was allowed to react for 15–16 h in the dark at room temperature. The radical solution was diluted with acetic acid (20 mM, pH 4.5) to obtain a solution showing an absorbance of 0.70 ± 0.02 at a wavelength of 734 nm. ABTS^•+^ solution (2 mL) was added to 200 μL of diluted sample (1 mg/mL). Absorbance was read, after 15 min, at 734 nm. The percentage decrease of absorbance against a blank sample (distilled water) was calculated. A standard curve was recorded using different concentrations of Trolox in the 0–10 μM range. The results were expressed as Trolox equivalents (mmol Trolox/100 g dry weight).

#### Hydroxyl Radical Scavenging Assay

For the determination of the site-specific and non-site-specific hydroxyl radical (^•^OH) scavenging activity, the methods described by Taghouti et al. (52) were used. Briefly, to 500 μL of sample solution (1 mg/mL), equal volumes (100 μL) of deoxyribose, iron chloride (1 mM), EDTA (1 mM), ascorbic acid (1 mM), and hydrogen peroxide (H_2_O_2_ 10 mM) were added. A phosphate buffer solution (400 μL; 20 mM, pH 7.4) was added to the mixture. After incubation at 37°C for 1 h, 1.5 mL TBA (in 10% TCA) was added, and the tubes were incubated for 15 min at 100°C. Absorbance was measured at 532 nm against a blank containing the above mixture without sample. The hydroxyl radical scavenging activity was defined as the percentage inhibition of the malonaldehyde production. A test without EDTA was also performed to evaluate the anti-radical scavenging activity of sample expressed as chelating ability. For this assay, the procedure is identical to that described above, but omitting the EDTA addition.

#### Nitric Oxide Radical Scavenging Assay

The nitric oxide radical scavenging activity was measured by monitoring the inhibition of nitric oxide generated from sodium nitroprusside, following the protocol of Sreejayan and Rao (53) with some modifications. Sodium nitroprusside solution (5 mM) was prepared in phosphate buffer (0.1 M H_3_PO_4_; pH 7.4) and oxygenated by purging air for 15 min. To 4.5 mL of this solution, 0.5 mL of sample solution (1 mg/mL) was added, and the mixture was incubated at 35°C in a water bath for 2 h. An aliquot of this solution (1 mL) was mixed with 1 mL of Griess reagent (5% H_3_PO_4_, 1% sulfanilamide, 0.1% *N*-alpha-naphthyl-ethylenediamine in water) and, after 3 min, the absorbance was measured at 546 nm. Standard solutions of sodium nitrite treated with the Griess reagent in the same way as the samples were used to quantify the radicals formed. NO scavenging activity was expressed as inhibition percentage; the blank consisted of nitroprusside solution without the sample.

### Determination of Total Dietary Fibre

For the determination of ISC and ISB dietary fibre content a modification of the AOAC Method 985.29 (54) was performed, by using the Megazyme Total Dietary Fibre kit (K-TDR-100A). Briefly, samples after dissolution (20 mg/mL of MES-TRIS buffer) were incubated in a shaking water bath firstly with 50 μL of heat stable α-amylase for 35 min, at 80°C, at pH 8.0, and then with 100 μL of protease at 60°C during 30 min. After adjusting pH to 4.1–4.5, the products were further hydrolysed with 200 μL of amyloglucosidase at 60°C during 30 min. This procedure allowed non-resistant starch and maltodextrin to solubilize and hydrolyse to D-glucose by the combined action of the two enzymes. The amount of glucose released was determined by high-performance anion-exchange chromatography (HPAEC, ICS-3000, Dionex) with a CarboPac PA-20 (Dionex), using rhamnose as internal standard. The non-resistant starch and maltodextrins were quantified by the amount of glucose released expressed as polymers.

### *In vitro* Cell-Based Assays

#### Cell Culture

Three cell lines were used: Caco-2 (human colorectal adenocarcinoma cell line; Cell Lines Services (CLS), Eppelheim, Germany), HepG2 (human hepatoma cell line; ATCC, Rockville, MD) and RAW 264.7 (murine macrophage cell line; CLS, Eppelheim, Germany). The three cell lines were maintained in Dulbecco's Modified Eagle Medium (DMEM), containing 4.5 g/L glucose, supplemented with 10% (v/v) foetal bovine serum (FBS), 2 mM l-glutamine, and antibiotics (100 U/mL penicillin and 100 μg/mL streptomycin), at 37°C in a 5% CO_2_ atmosphere under controlled humidity.

#### Cell Viability/Cytotoxicity Assay

The cytotoxicity/safety of both ISB EtSn and ISC EtSn melanoidins was evaluated by performing a dose-dependent exposure assay, in which cells were exposed to a set of ISB EtSn and ISC EtSn melanoidins different concentrations (up to 1 mg/mL). Dilutions were made in FBS-free culture media, under aseptic conditions (laminar flow chamber). Adherent cell lines (Caco-2 and HepG2) were handled as previously described by Andreani et al. (55) and Silva et al. (56). Briefly, confluent cells were detached with Trypsin-EDTA at 0.05 %, counted, resuspended in culture medium (at 5.0 × 10^4^ cells/mL), seeded in flat bottom 96-well microplates (5.0 × 10^3^ cells/well) and cultured for 24 h (for cell adhesion). RAW 264.7 cells were manipulated as described in (55, 57). Briefly, cells were scraped from the flask and subjected to the same procedures as described below. After seeding period, culture media was removed and replaced by test solutions, and cells were further incubated for 24 or 48 h (different assays). After these periods, the test solutions were removed and immediately replaced by cell viability indicator solution (FBS-free culture medium supplemented with Alamar Blue (AB; Alfagene, Invitrogen, Portugal) solution (10%, v/v), 100 μL/well). After 5 h incubation with AB, absorbances were read at 570 nm (AB reduced form; resorufin) and at 620 nm (AB oxidised form; resazurin). Cell viability data were analysed by calculating the percentage of AB reduction (according to the manufactures recommendation) over the percentage of control (untreated cells), as previously reported (55). All media and reagents used for cell culture were from Gibco (Invitrogen, Life Technologies, Portugal).

#### Assessment of Anti-inflammatory Activity

The anti-inflammatory activity of ISB EtSn and ISC EtSn melanoidins was assessed in RAW 264.7 cells [maintained as described in previous subsection and as in (36)]. Cells seeded (at 2.0 × 10^5^ cells/mL) in 96-well culture plates (100 μL/well) were cultured for 48 h for adherence and stabilisation, before being exposed to EtSn melanoidin solutions (at different non-cytotoxic concentrations). Test solutions were prepared in two sets: (i) in FBS-free culture medium and (ii) in FBS culture medium supplemented with 1 μg/mL lipopolysaccharide (LPS), the inflammatory stimuli. LPS binds to Toll-like receptor (TLR) in macrophage plasma membrane and induces the production of nitric oxide (NO) that is released into the incubation media. Thus, cells were incubated for 24 h with ISB EtSn and ISC EtSn melanoidins solutions, both in the absence and in the presence of LPS. The nitrite production (end product of NO metabolism) reflecting NO production was measured in the cells' supernatants by a colorimetric reaction using Griess reagent. Briefly, 50 μL of supernatant from each well were transferred into a new 96-well plate and subsequently mixed with 50 μL of Griess reagent [0.1% (w/v) *N*-(1-naphthyl)-ethylenediamine dihydrochloride and 1% (w/v) sulfanilamide prepared in 5.0% (w/v) H_3_PO_4_ (v/v)], and incubated for 15 min in the dark before reading the absorbance at 550 nm, using a Multiskan EX microplate reader (MTX Labsystems, Inc., Bradenton, FL, USA). Nitrite produced by cells was quantified by means of a standard curve obtained using serial dilutions of NaNO_2_ (0 to 100 μM) in fresh FBS-free culture medium, prepared *de novo* in each independent assay. Results are expresses as percentage of control (nitrite production by the positive control cells, i.e., LPS-stimulated cells in the absence of EtSn melanoidins) set to 100%, that is, 0% of anti-inflammatory effect, as mean ± S.D. (*n* = 3 independent assays, each independent assay in quadruplicates) (56, 57).

### *In vitro* Assessment of Enzymatic Activity Inhibition

The potential role of EtSn melanoidins in inhibiting the activity of several key enzymes that regulate a variety of biological functions, such as carbohydrate digestion (*via* α-amylase and α-glucosidade), acetylcholine (neurotransmitter) degradation after exocytosis (*via* acetylcholinesterase, AChE), melanin and neuromelanin synthesis (*via* tyrosinase) or elastin degradation (*via* elastase) was assessed *in vitro* using already described procedures (52). EtSn melanoidins were tested at the concentration range 0.0625–1 mg/mL (in triplicates) to assess the capacity to inhibit acetlycholinesterase, tyrosinase, elastase, α-amylase, and α-glucosidase; all assays were performed in 96-wellplates (PowerWave XS2, BioTek) as described in (54). As positive controls of enzyme inhibition, acarbose (1 mg/mL) was used as specific inhibitor of α-amylase and of α-glucosidase and kojic acid (1 mg/mL) was used as specific inhibitor of tyrosinase. Non-specific enzymatic inhibition of acetylcholinesterase and of elastase were performed by addition of methanol instead of the substrate solution.

### Statistical Analyses

All *in vitro* measurements were performed at least in triplicate (*n* = 3), cell viability and anti-inflammatory assays were performed at least in 3 independent assays (4 measurements per assay) and values were expressed as media ± standard deviation (SD). The results were subjected to *t*-Student test, analysis of variance (ANOVA), one-way or two-way (depending on the assay), and a multiple range test (Tukey's test) using SPSS statistic 21.0 software package (SPSS Inc., Chicago, USA). Significant differences between the biological activity of the compounds and combinations assessed were set at *p* < 0.05.

## Results and Discussion

### Chemical Composition of Melanoidins Isolated From Instant Coffee and Instant Barley

As reported in Table 1, instant coffee (ISC) and instant barley (ISB) contained significant amounts of high molecular weight material (HMWM) which represented about 35 and 56% of the dialyzed material, respectively, in line with the values present in the literature (32, 58). The HMWM isolated from ISB, despite its brown colour, was largely composed by carbohydrates (93%), mainly glucose (88%) and smaller amounts of other sugars such as xylose, arabinose, galactose, and rhamnose. This chemical composition was significantly different from the HMWM isolated from ISC that was composed by 59% of carbohydrates, mainly galactose (38%) and mannose (13%), and lower amounts of arabinose and glucose. This distinct sugar composition reflected the different polysaccharide composition of green coffee beans and barley seeds, with the first one being mainly composed by (galacto)mannans and type II arabinogalactans (5) and barley seeds being mainly composed by starch, β-glucans, and arabinoxylans (59). The sugar composition of ISC HMWM showed that it contained more type II arabinogalactans when compared to normal coffee drink and less HMWM galactomannans (24, 26, 48, 60, 61), in accordance with previous studies on ISC HMWM (62). The carbohydrate content of the ISB was also in line with previous results described in literature (32).

**Table 1:** Yield and sugar and phenolic composition of melanoidins and melanoidins population isolated from instant soluble coffee and instant soluble barley.

	**Yield**	**Sugars (g/100 g)**								**Phenolic Compounds (g caffeic acid equivalents/100 g)**			
		**Fuc**	**Rha**	**Ara**	**Xyl**	**Gal**	**Glc**	**Man**	**Total**	**Adsorbed**	**Esterified**	**Condensed**	**Total**
**Coffee**													
HMWM	34.8	0.41 ± 0.19	0.92 ± 0.11	3.39 ± 0.05	0.64 ± 0.25	37.97 ± 1.7	2.05 ± 0.45	13.47 ± 0.44	58.84 ± 3.1	n.d	0.50 ± 0.04	16.6 ± 0.4	17.1 ± 0.4
Et50	8.3	0.61 ± 0.05	0.42 ± 0.04	0.98 ± 0.03	0.19 ± 0.05	10.24 ± 0.02	1.81 ± 0.04	56.02 ± 0.04	70.26 ± 2.3	n.d	0.15 ± 0.04	6.5 ± 0.3	6.7 ± 0.3
Et75	49.5	0.31 ± 0.00	0.70 ± 0.05	3.35 ± 0.06	0.29 ± 0.04	47.44 ± 1.3	1.74 ± 0.11	8.79 ± 0.14	62.61 ± 1.7	n.d	0.22 ± 0.03	3.8 ± 0.2	4.0 ± 0.2
EtSn	42.3	0.34 ± 0.04	1.01 ± 0.09	3.38 ± 0.03	0.34 ± 0.04	30.48 ± 0.84	1.30 ± 0.14	6.10 ± 0.15	42.98 ± 1.2	n.d	0.21 ± 0.0	14.4 ± 0.2	14.6 ± 0.2
**Barley**													
HMWM	55.8	0.48 ± 0.04	0.89 ± 0.54	0.35 ± 0.01	1.03 ± 0.36	0.99 ± 0.09	88.48 ± 2.3	0.64 ± 0.18	92.86 ± 3.5	n.d	0.002 ± 0.002	8.3 ± 0.3	8.3 ± 0.3
Et50	3.3	–	–	–	–	–	–	–	–	–	–	–	–
Et75	2.6	0.05 ± 0.02	0.09 ± 0.05	0.04 ± 0.00	0.07 ± 0.01	0.09 ± 0.02	7.31 ± 0.48	0.05 ± 0.02	7.70 ± 0.38	n.d	n.d	2.2 ± 0.2	2.2 ± 0.2
EtSn	94.2	0.43 ± 0.11	0.66 ± 0.05	0.39 ± 0.09	0.85 ± 0.40	0.72 ± 0.09	76.13 ± 5.0	0.97 ± 0.22	80.15 ± 4.3	n.d	n.d.	3.0 ± 0.3	3.0 ± 0.3

*n.d., not detected; –, not analysed due to low amount of material obtained*.

The HMWM isolated from ISC and ISB presented significant amounts of associated phenolic compounds, as measured by the Folin-Ciocalteu assay (Table 2), with the ISC—HMWM isolated presenting almost twice the amount of phenolic compounds compared to those present in ISB. The same trend was observed for the *ortho*-diphenols content (Table 2). The amount of total polyphenols present in each HMWM were in line with those previously described in literature (24, 28, 32).

**Table 2:** ISC and ISB sum phenolic composition and antioxidant activity.

	**Instant coffee**	**Barley coffee**	***t*-test**
Total phenols (g/100 g)	18.5 ± 3.2	10.1 ± 2.1	*p* <0.0048
*Ortho*-diphenols (g/100 g)	7.2 ± 0.1	4.3 ± 0.2	*p* <0.0001
ABTS^•+^ (g Trolox/100 g)	329.2 ± 1.6	123.5 ± 10.0	*p* <0.0001
^•^OH with EDTA (% inhibition)^a^	38.3 ± 2.9	49.7 ± 3.3	*p* <0.0020
^•^OH without EDTA (% inhibition)^a^	41.1 ± 3.7	50.5 ± 0.6	*p* <0.0024
NO^•^ (% inhibition)^a^	56.9 ± 1.1	25.7 ± 4.8	*p* <0.0001

a *% of inhibition for 1 mg/mL extract*.

Coffee HMWM has been shown to contain esterified, glycosidic-linked and condensed phenolic compounds (29), nevertheless these phenolic compounds could also be present adsorbed to the HMWM (25, 28, 63); therefore the amount of adsorbed, esterified, and condensed phenolic compounds were determined by the profiling scheme previously developed (28). No adsorbed phenolic compounds could be detected by direct analysis of the HMWM solutions by RP-HPLC. The application of alkaline saponification allowed to identify 0.50 g of caffeic acid equivalents/100 g (2.78 mmol of phenolic compounds/100 g), in line with previous results (24, 27–29, 33, 35, 64–66). On the other hand, the ISB-HMWM presented smaller amounts of phenolic compounds released by alkaline saponification. It was known that barley arabinoxylans contained esterified phenolic compounds that include *p*-coumaric, ferulic acid, and the ferulic acid dimers (59); therefore, these phenolic compounds could represent the fraction esterified with arabinoxylans, although the amount of arabinoxylans in the ISB-HMWM was low (Table 1). Alkaline fusion of both HMWMs allowed to release phenolic compounds either from ISC and ISB with ISC presenting a significant higher amount when compared to ISB. The amount determined was almost twice for the ISC when compared to ISB in line with the results obtained by Folin-Ciocalteu assay (Table 2). The difference in incorporated phenolic compounds was in line with the different phenolic compounds content and nature in coffee (67) and barley (68).

**Figure 1:**
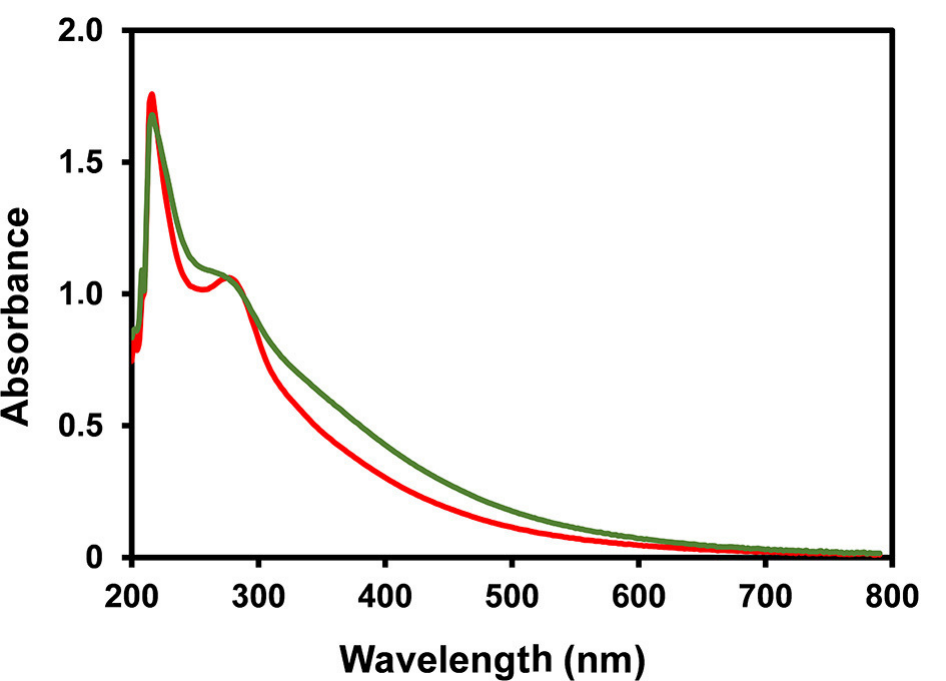
UV-Visible spectra of EtSn ISC melanoidin population (green line) and of ISB melanoidin population (red line).

The HMWM from coffee infusions has been shown to be composed by a different population concerning their polysaccharide composition with galactomannans enriched material being insoluble in 50% of ethanol and arabinogalactans in 75% ethanol and most of the brown coloured compounds being soluble in 75% ethanol solution (48, 60, 61). The ethanol fractionation of both HMWMs was performed and differences in solubility were registered. In fact, ISB-HMWM was almost exclusively soluble in 75% ethanol solution while ISC-HMWM presented a typical distribution of the HMWM previously registered in literature (Table 1). The chemical composition concerning the polysaccharides present in each fraction were in accordance with coffee literature (48, 60, 61), nevertheless the amount of material recovered in each fraction was significantly different from coffee drinks where the abundance of the Et50 fraction, rich in melanoidin and melanogalactomannan populations, was far more abundant than the Et75 fraction composed by type II arabinogalactans and melanoarabinogalactans (26, 48, 60, 61). On the other hand, almost all the material obtained from ISB was soluble in 75% ethanol solution being again almost exclusively composed by glucose as observed for the HMWM (Table 1). ISC and ISB melanoidin populations, soluble in 75% ethanol presented a different K_mix405nm_ (Figure 1) and contribute to the colour of the initial material. K_mix405nm_ registered for EtSn material from ISC was higher than that observed for the same fraction isolated from ISB (1.76 vs. 0.54) although the EtSn fraction from ISB accounted for 53.5% of the barley coffee brown colour and the same fraction from coffee only for 23.0% of the ISC brown colour. These results were in accordance with those reported by Tagliazucchi et al. (32) who observed that ISB melanoidins presented a lower K_mix_ than ISC melanoidins.

### Dietary Fibre Content of EtSn Fraction From ISC and ISB

Considering that the main component of barley melanoidins was glucose and that barley was rich in starch containing also significant amounts of β-glucans (59), the EtSn melanoidin population of ISB could be composed by starch transformation products (69). Therefore, the ISB EtSn melanoidins accounting for 95% HMWM was subjected to hydrolysis using the Dietary Fibre protocol (54) but with a slight modification consisting in quantifying the glucose released by anion-exchange chromatography. Only 46% of the glucose determined by acid hydrolysis was released after treatment with amylase, protease, and amyloglucosidase, showing that about half of the glucose present in the EtSn melanoidin population was resistant to enzymatic hydrolysis and was probably derived from β-glucans or resistant starch. No glucose was detected after the hydrolysis of ISC EtSn melanoidins, being this fraction resistant to the alpha-amylase, protease, and amiloglucosidase digestion, as expected from the known polysaccharide composition of these melanoidins (Table 1).

### *In vitro* Antioxidant and NO Scavenging Activity

The *in vitro* antioxidant activity was evaluated by measuring ISC and ISB EtSn melanoidins capacity to scavenge ABTS^•+^ radical, the site specific and non-specific hydroxyl radical (^•^OH) and the NO radical (Table 2). Results indicated that ISC EtSn melanoidin population had a significantly higher ABTS^•+^ scavenging activity than ISB EtSn melanoidin population (Table 2), in accordance with literature (32, 33, 70). The higher antioxidant activity was in line with the higher phenolic content associated with the EtSn ISC melanoidins when compared to the EtSn ISB melanoidins [Table 1; (71)]. The same trend was also observed for the NO radical scavenging activity (Table 2). Conversely, ISB EtSn populations had significantly higher scavenging activity against the site-specific and non-specific ^•^OH radicals. This result was quite surprising as it was shown that coffee melanoidins had a higher iron-chelating ability toward free Fe^2+^ ions than barley melanoidins (32), but the different source of ISC and ISB melanoidins used in this work, the fact that only the EtSn melanoidins population was studied using also different tests used could explain the different results here obtained.

### Evaluation of Cytotoxic Effect of Instant Barley and Instant Coffee EtSn Melanoidins

**Figure 2:**
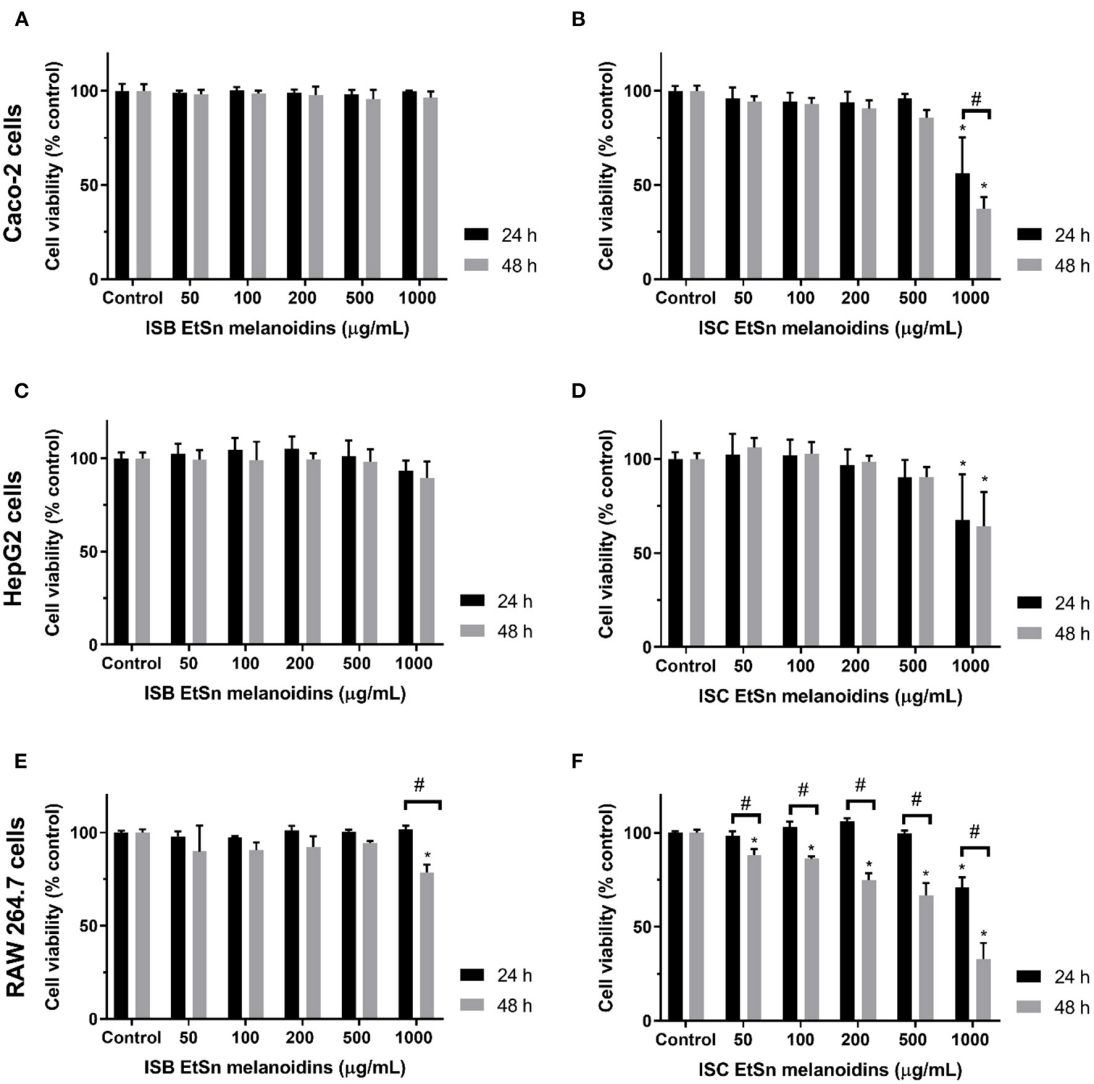
Effect of ISC and ISB EtSn melanoidins on cell viability. Caco-2 **(A,B)**, HepG2 **(C,D)**, and RAW 264.7 **(E,F)** cells were exposed to different concentrations (up to 1 mg/mL, as denoted) of ISC and ISB EtSn melanoidins for 24 h (black bars) or 48 h (grey bars). Cell viability was assessed using Alamar blue indicator, results are expressed as percentage of control (unexposed cells) and data represent mean ± S.D. (*n* = 3 independent experiments, each one with 4 replicates). (*) denotes statistical differences, comparing to respective control. (#) denotes statistical difference, comparing the same concentration between time-points.

Although melanoidins have been described as having positive biological activities, elevated doses of these dietary compounds may also be cytotoxic and mutagenic (40). Therefore, EtSn melanoidin populations from ISC and ISB were evaluated concerning the cytotoxic effect in three different cell lines, i.e., human colon (Caco-2), human hepatic (HepG2), and murine macrophage (RAW 264.7). Cells were exposed to a 0.05–1 mg/mL range of ISC and ISB EtSn melanoidin, for 24 and 48 h and the results of cell viability were shown in Figure 2. ISB EtSn melanoidin populations did not significantly affect Caco-2 (Figure 2A) and HepG2 (Figure 2C) cell viability in all the concentration range tested, although a slight reduction in cell viability was registered at 1,000 μg/mL, especially after 48 h exposure (*p* > 0.05). The effect of ISB EtSn melanoidin populations on RAW 264.7 cells was identical (Figure 2E), but when tested at 1,000 μg/mL, after 48 h exposure, a significant reduction of cell viability, of about 20% (cell viability was 78.7 ± 4.1% of control), was observed (*p* < 0.05). Considering the effect of ISC EtSn melanoidin populations, it was very similar to that of ISB EtSn melanoidins in Caco-2 (Figure 2B) and HepG2 cells (Figure 2D), with the exception of the highest tested concentration (1,000 μg/mL) which significantly reduced the cell viability both after 24 and 48 h of exposure (*p* < 0.05). At 1,000 μg/mL, cell viability reduction was slightly more marked for Caco-2 (~40 and ~60% reduction, at 24 and 48 h, respectively) than for HepG2 cells (~33 and ~35% reduction, at 24 and 48 h, respectively). By exposing for 48 h RAW 264.7 cells to ISC EtSn melanoidin populations, a dose-dependent reduction on cell viability was registered with an IC_50_ = 727 ± 120 μg/mL (Table 3); concentrations up to 500 μg/mL, in contact with cells for 24 h, did not significantly reduce cell viability after 24 h of exposure (*p* > 0.05), but, 1,000 μg/mL reduced cell viability to 70.9 ± 5.4% (Figure 2F). These data indicated that ISB EtSn melanoidin populations were non-toxic for the three cell lines tested up to 1,000 μg/mL while ISC melanoidinic fraction was non-toxic for Caco-2 and HepG2 cells up to 500 μg/mL. Concerning the effect of ISC EtSn melanoidin populations on RAW 264.7 cells, it resulted in a dose-dependent cytotoxic activity (Figure 2F and Table 3), at longer exposure time. This effect might result from the higher amount of total phenol compounds present in EtSn melanoidin populations compared to ISB EtSn melanoidins (Table 2).

**Table 3:** IC_50_ values of ISC and ISB EtSn melanoidin populations in different cell lines and exposure times.

		**IC**_**50**_ **(μg/mL)**	
		**24 h**	**48 h**
Caco-2	Coffee	>1,000	776 ± 27
	Barley	>>1,000	>>1,000
HepG2	Coffee	>1,000	>1,000
	Barley	>>1,000	>>1,000
RAW 264.7	Coffee	>>1,000	727 ± 121
	Barley	>>1,000	>>1,000

### Anti-inflammatory Activity of Instant Barley and Instant Coffee EtSn Melanoidins

**Figure 3:**
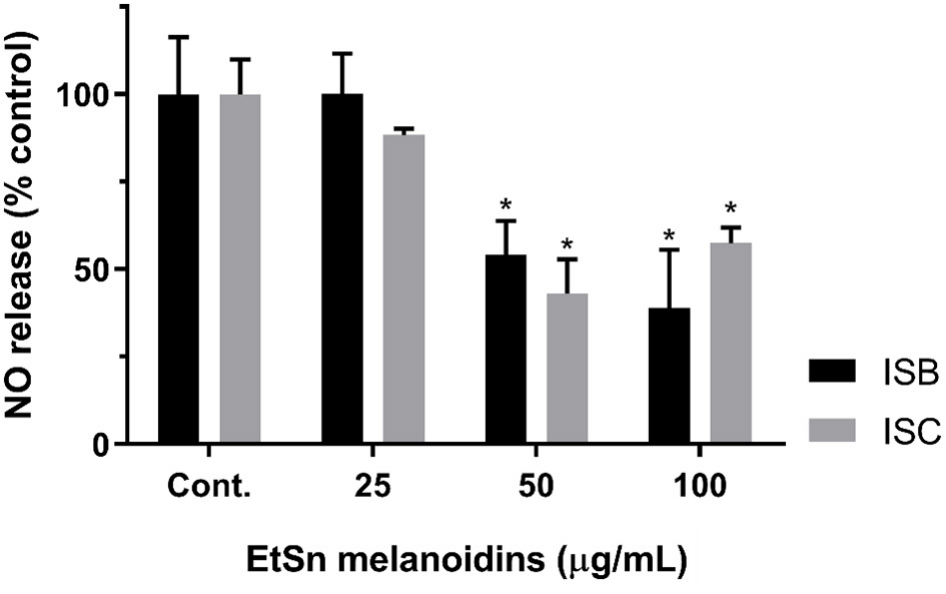
Anti-inflammatory potential of ISC and ISB EtSn melanoidins. RAW 264.7 cells were exposed to different concentrations of ISB (black bars) and of ISC (grey bars) EtSn melanoidins (0, 25, 50, and 100 μg/mL) in the presence of LPS (1 μg/mL), please see methods for details. The supernatant was collected 24 h later and NO production was assayed using the Griess reagent. Results are expressed as percentage of control (LPS exposed cells) and data represent mean ± S.D. (*n* = 3 independent experiments, each one with 4 replicates). (*) denotes statistical differences, comparing to respective control.

Considering that ISC and ISB EtSn melanoidin populations presented significant amounts of non-adsorbed phenolic compounds and that both were not toxic for RAW 264.7 cells when tested up to 200 μg/mL, concentrations up to 100 μg/mL (to assure that reduction in NO release was not related to changes in cell viability) were used to assess their efficacy in reducing the NO release from LPS-stimulated macrophage cells. Figure 3 showed the NO release from LPS-stimulated macrophage cells incubated with both EtSn melanoidin populations. Both melanoidin fractions induced a strong inhibitory action with higher activity registered for ISB fraction than ISC (at 100 μg/mL, NO release was ~39 and ~57% for ISB and ISC, respectively; *p* > 0.05). These results indicated that the inhibitory action could not to be correlated with the amount of phenolic compounds present in the melanoidins as ISC contained a significantly higher amount of total phenolic compounds when compared to ISB, but probably the nature of the phenolic compounds or the polysaccharide composition of these fractions could affect this activity. In addition, the direct inhibition of NO radicals (by radical scavenging; Table 2) was lower for ISB than ISC (Table 2) and therefore the differences observed for both melanoidin fraction in inhibiting the LPS-stimulated NO production might rather be through a cellular effect, either at the TLR level or downstream in its signalling cascade. Indeed, coffee roasting products have been shown to negatively regulate the transcription factor, nuclear factor kappaB (NF-κB), after LPS-stimulation of RAW 264.7 cells, which resulted in decreased NO release (72), as here observed (Figure 3).

Maillard Reaction Products (MRPs) in many thermally processed Maillard Reaction mixtures provided bioactive properties, such as quenching reactive oxygen species (ROS) and reactive nitrogen species (RNS), which was reported to be negatively correlated with colour change, but positively correlated with sugar loss (73). Anti-inflammatory effect of MRPs derived from heated aqueous glucose–lysine (Glu–Lys) was reported in Caco-2 cells induced by interferon-γ and phorbol ester by the ability to inhibit nitric oxide (NO) and interleukin 8 (IL-8) production (74). Improvement of anti-oxidative status and anti-inflammatory activity promoted by extracts obtained from *Coffea arabica* green coffee was reported (75), with a decreasing effect with the increasing of roasting process. In Table 2 and in Figure 3 we reported that low concentrations (100 μg/mL) of EtSn melanoidins, clearly produced anti-inflammatory effect by reducing LPS-stimulated NO release.

### *In vitro* Assessment of the Neuroprotection, Anti-ageing, and Antidiabetic Activity of Instant Coffee and Instant Barley EtSn Melanoidins

The effect of ISC and ISB EtSn melanoidins on the activity of key enzymes was assessed and results were presented in Table 4. The activity of AChE was assessed since several neurodegenerative diseases result from of a decline in neurotransmitter production and secretion by neurons, as is the case of Parkinson's and Alzheimer's diseases, in which reduced brain acetylcholine (ACh) levels was a common biochemical change (76). Beyond the brain, cholinergic signalling plays a relevant role on most peripheral organs, such as controlling muscular contraction, gland secretion, inflammation (77), and recently particular attention is given to its role in inflammatory bowel diseases (78), and others. Restoration of ACh levels was pharmacologically achieved by drugs that inhibited AChE [and/or butrylcholinesterase (BChE)]; however, more recently, natural approaches were also suggested in order to prevent and/or ameliorate diseases whose symptoms are associated with a cholinergic decline. In Table 4, results show that ISC when tested at 1 mg/mL inhibited ~58% of AChE activity while ISB only inhibited ~29%, demonstrating the neuroprotective potential of these products. The higher AChE inhibitory capacity registered for ISC might also be associated with the higher content in TPC and *ortho*-diphenols in comparison to ISC (Table 2), as polyphenol rich extracts were reported to inhibit AChE (32). Tyrosinase, beyond being responsible for the skin and hair melanin synthesis, was also implicated in neuromelanin formation in human brain (79). Neuromelanin, by interacting with many toxicants (e.g., heavy metals, pesticides), may play an important role in both the initiation and progression of neurodegeneration (79). Thus, products that inhibited tyrosinase activity could have potential as neuroprotective agents but also have applicability in cosmetic industry as agents with skin lightening properties. As observed in Table 4, both EtSn melanoidins at 1 mg/mL had high tyrosinase inhibitory capacity. As the bioavailability of melanoidins in brain tissue might be very low, their potential role in reducing the neuromelanin formation is small, however these compounds have cosmeceutical potential as skin lightening agents. Concerning the potential to inhibit elastase, only ISC EtSn melanoidins inhibited ~10% of the enzyme activity (Table 4), thus presenting a low to moderate anti-ageing effect. The enzymes α-amylase and α-glucosidade hydrolyse dietary carbohydrates to simple sugars and during digestion contribute to the bioavailability of glucose to intestinal absorption; thus, molecules that inhibit the function of these enzymes could contribute to reduce glucose absorption and therefore hyperglycaemia in type-2 diabetic patients. Based on these considerations, the anti-diabetic potential of EtSn melanoidins was also assessed. As shown in Table 4, both melanoidin fractions at 1.0 mg/mL, did not inhibit α-amylase activity and poorly inhibited α-glucosidase activity (~5%), demonstrating that they had no anti-diabetic potential. Comparing ISC and ISB EtSn melanoidins, ISC showed better enzyme function inhibitory activity than ISB (Table 4), which positively correlated with the TPC and *ortho*-diphenols content (Table 2).

**Table 4:** *In vitro* assessment of ISC and ISB EtSn melanoidin population inhibitory activity (%) against acetylcholine esterase (AChE), tyrosinase, elastase, α-glucosidase, and α-amylase.

**Extract**	**AChE**	**Tyrosinase**	**Elastase**	**α-Glucosidase**	**α-Amylase**
Coffee	58 ± 5	89 ± 1	10 ± 4	7 ± 3	ND
Barley	29 ± 1	87 ± 1	ND	4 ± 3	ND

*Extracts prepared at 1.0 mg/mL. Values are presented as means SD; n = 3. ND: not detected*.

## Conclusion

Instant soluble barley HMWM presented significantly higher polysaccharide content (93 vs. 59%) and lower amount of phenolic compounds (8 vs. 17%), mainly present in condensed structures, when compared to instant soluble coffee HMWM. This higher content of phenolic compounds in instant soluble coffee HMWM may explain also the higher *in vitro* scavenging activity against ABTS and NO radicals when compared to instant soluble barley. Nevertheless, instant soluble barley melanoidins showed a higher inhibition of NO production by LPS-stimulated macrophages at higher concentrations, showing that the inhibitory effects on NO production were unique for each melanoidin. Both melanoidins, by inhibiting several key enzymes, showed high neuroprotective, moderate antiaging effect and low to moderate antidiabetic effect. Thus, consumption of a diet rich in instant soluble barley and instant soluble coffee melanoidins could reduce the oxidative stress and the levels of inflammatory mediators, contributing to protective effects against chronic inflammatory diseases, such as inflammatory bowel diseases, among others.

## Data Availability Statement

The original contributions presented in the study are included in the article/supplementary material, further inquiries can be directed to the corresponding author.

## Author Contributions

AS, AP, and FN contributed to conception and design of the study. SA, DA, and LF organised the database. AS and FN performed the statistical analysis and wrote the first draft of the manuscript. SA, LF, CS, and LF wrote sections of the manuscript. All authors contributed to manuscript revision, read, and approved the submitted version.

## Funding

This research was supported by funds from the Portuguese Science and Technology Foundation (FCT), Ministry of Science and Education (FCT/MEC) through national funds, co-financed by FEDER, under the projects UIDB/04033/2020 (CITAB), UIDB/00616/2020, and UIDP/00616/2020 (CQ-VR).

## Conflict of Interest

The authors declare that the research was conducted in the absence of any commercial or financial relationships that could be construed as a potential conflict of interest.

## Publisher's Note

All claims expressed in this article are solely those of the authors and do not necessarily represent those of their affiliated organizations, or those of the publisher, the editors and the reviewers. Any product that may be evaluated in this article, or claim that may be made by its manufacturer, is not guaranteed or endorsed by the publisher.
